# The unpredictable journeys of spreading, sustaining and scaling healthcare innovations: a scoping review

**DOI:** 10.1186/s12961-019-0482-6

**Published:** 2019-09-13

**Authors:** Élizabeth Côté-Boileau, Jean-Louis Denis, Bill Callery, Meghan Sabean

**Affiliations:** 1 Health Sciences Research, Faculty of Medicine and Health Sciences, University of Sherbrooke, Quebec, Canada; 2 Charles-Le Moyne – Saguenay–Lac-Saint-Jean Research Center on Health Innovations, Quebec, Canada; 3 Doctoral Award Fellow from Quebec’s Fonds de recherche du Québec – Santé (FRQS), Quebec, Canada; 4 Health Standards Organization, Ottawa, Canada; 5 Health Administration Department, School of Public Health, University of Montreal, Quebec, Canada; 6 University of Montreal Hospital Research Center, Quebec, Canada; 7 Canada Research Chair (Tier I) holder on Health system design and adaptation (Canadian Institutes of Health Research), Montreal, Canada; 8 Canadian Foundation for Healthcare Improvement, Corporate Strategy and Program Development, Ottawa, Canada

**Keywords:** Innovation, spread, sustainability and scale-up, definitions, mechanisms, support conditions, large-scale system transformation, quality improvement

## Abstract

Innovation has the potential to improve the quality of care and health service delivery, but maximising the reach and impact of innovation to achieve large-scale health system transformation remains understudied. Interest is growing in three processes of the innovation journey within health systems, namely the spread, sustainability and scale-up (3S) of innovation. Recent reviews examine what we know about these processes. However, there is little research on how to support and operationalise the 3S. This study aims to improve our understanding of the 3S of healthcare innovations. We focus specifically on the definitions of the 3S, the mechanisms that underpin them, and the conditions that either enable or limit their potential. We conducted a scoping review, systematically investigating six bibliographic databases to search, screen and select relevant literature on the 3S of healthcare innovations. We screened 641 papers, then completed a full-text review of 112 identified as relevant based on title and abstract. A total of 24 papers were retained for analysis. Data were extracted and synthesised through descriptive and inductive thematic analysis. From this, we develop a framework of actionable guidance for health system actors aiming to leverage the 3S of innovation across five key areas of focus, as follows: (1) focus on the why, (2) focus on perceived-value and feasibility, (3) focus on what people do, rather than what they should be doing, (4) focus on creating a dialogue between policy and delivery, and (5) focus on inclusivity and capacity building. While there is no standardised approach to foster the 3S of healthcare innovations, a variety of practical frameworks and tools exist to support stakeholders along this journey.

## Background

It is difficult to understand how innovations circulate in highly institutionalised and rapidly changing environments such as health systems [1–5]. Health systems in various jurisdictions are slow to adapt, innovate and improve at a sufficient pace [6–8]. According to Health Quality Ontario “*… fewer than 40% of healthcare improvement initiatives successfully transition from adoption to sustained implementation that spreads to more than one area of an organization*” ([7], p. 4). This can be challenging to healthcare communities intent on increasing the impact of innovations within and beyond jurisdictions [8, 9]. The innovation journeys that would enable improvement in local settings to expand and bring about large-scale health system transformation remains something of a black box [10, 11]. A growing body of research in health systems focuses on three specific processes as potential levers to accelerate improvement and innovation, namely the spread, sustainability and scale-up (hereafter referred to as the 3S) of healthcare innovations [12–16].

The literature on the 3S of healthcare innovations highlights that these processes unfold along a continuum [17–23], where progress is enabled or challenged by a set of unpredictable dynamics, contextual factors and organisational processes [24–28]. The growing interest in the 3S reflects a need to respond to the challenge of increasing the innovative capacities of health systems and organisations. However, against the promise of the 3S of innovation, scholars stress that innovation is, in effect, a journey, which is unpredictable in nature and involves social, dynamic and non-linear processes [29–38]. Thus, there seems to be an emerging tension in the literature between, on the one hand, the idea that the journeys innovation takes through the 3S can be grasped, supported and achieved by means of a structured approach, and on the other, the idea that neither the journeys of innovation nor their effects can be predicted. In order to reconcile this tension, we consider that the social, dynamic and iterative characteristics of innovation journeys are themselves the structuring pillars of innovations. Hence, while paying attention to the social dynamics that underlie innovation journeys through the 3S may not enable us to predict their course or effects, it may bring us closer to discovering the sources of significant changes that appear along the way.

While the structural changes commonly used in healthcare improvement efforts may help create a more receptive context for innovation, they do not appear sufficient to foster the 3S of healthcare innovations and achieve system transformation [39–41]. Large structural reorganisations generally fail to overcome the change-resistant nature of healthcare systems with regards to lasting improvement [42]. Other levers are needed to accelerate uptake of local innovations more systematically [40, 43–50]. These include engagement of front-line managers and providers in a culture of improvement, a focus on population needs, supportive policies and incentives, investment in organisational capacity, participation of patients and citizens, and evidence-informed decision-making [51–54].

This review aims to consolidate the evidence on the 3S of healthcare innovation to better understand how they work and the mechanisms and contextual conditions that enable complex health systems and organisations to increase uptake of innovations.

### The legacy of the diffusion of innovation model

Everett Rogers’ seminal research on the diffusion of innovations model (DIM) moved the field from technological determinism (i.e. improvements will inevitably be adopted) to a focus on social dynamics (i.e. social factors determine whether and how an improvement will be adopted) [16, 20–22, 55]. The innovation journey according to Rogers is a process of social exchange and construction in which meanings and values attributed to the innovation take form [56]. His work illustrates that it is not just the properties (relative advantage, compatibility, complexity, trialability and observability) of innovations that determine their diffusion [36, 56], but rather an aggregate set of factors associated with social relations and communication across networks [57, 58]. These include government regulations, social values promoted by various actors and human interactions around a given innovation [22, 59]. Indeed, the properties of an innovation will not have the same meaning and value for all actors within a given context, and communication among various individuals and groups within and across contexts influence the acceptability and dissemination of the innovation [59].

The DIM helps to understand the dynamics that take place in centralised diffusion systems as well as decentralised systems that recognise the agency of users in shaping an innovation [58]. However, the DIM does not focus on the mechanisms and enabling conditions for moving innovations from local to large-scale uptake within complex and highly institutionalised sectors such as healthcare. This paper aims to address this gap, in part by looking at the 3S of healthcare innovations within Rogers’ DIM perspective on the innovation journey.

## Methods

### Scoping review

A scoping review of the literature was undertaken between October 2016 and April 2017, commissioned by the Canadian Foundation for Healthcare Improvement (CFHI). The central research question was: How to facilitate the 3S processes of healthcare innovations? Booth’s five-stage process for scoping reviews [60] was employed, involving (1) an exploratory scoping search of existing reviews to get a sense of the volume and scope of available literature on the research topic in order to identify relevant databases and key search terms for the search strategy, (2) a search for relevant peer-reviewed articles and grey literature papers in these databases, using key search terms (both free-text and thesaurus terms), (3) a search for additional relevant articles by screening the bibliographies (reference lists) of all papers, (4) revision and modification of the initial search strategy to ensure that we included all articles potentially relevant to the research question, and (5) extraction, analysis and recording of data from all articles in the form of summary tables.

### Search strategy

We started by exploring 48 prior studies to develop our search strategy. We then used three search engines (EBSCOhost, ERIC, Google Scholar) and seven electronic databases (CINHAL, Academic Search Complete, Business Complete Source, PsycINFO, SocINDEX, MEDLINE, EconLit) to comprehensively search for articles, using the following key search terms: How to Spread OR How to Sustain* OR How to Scale AND Innov* AND health OR healthcare OR health organization* OR health system*. We identified 641 potentially relevant papers from grey and peer-review literature for the review. A two-stage screening process was used. The first stage consisted in reviewing articles by title and abstract, which resulted in 112 articles meriting further review. Papers were retained for inclusion if (1) abstracts included the word(s) spread* AND/OR sustain* AND/OR scale*, (2) papers were specific to the healthcare domain, (3) papers provided conceptual and/or empirical guidance on how to facilitate the 3S processes of healthcare innovation, and (4) papers represented OECD countries. A total of 18 papers met these criteria and were retained. Screening the bibliographies of these papers and hand searching and verification identified 26 additional papers that went on to full-text review, of which 7 met the above criteria and were retained, bringing us to a total of 25 articles for analysis. Finally, the documentation stage involved extracting, analyzing and summarising the following data from the 24 papers included in the review:

1. Authors and title
2. Research question/aim
3. Methodological design
4. Main process(es)
5. Definitions
6. Mechanisms
7. Enabling and limiting factors

### Data analysis

We used a two-phase analytical approach to extract and synthesise data from retained papers. First, a descriptive analysis was undertaken to categorise papers according to (1) grey literature or peer-reviewed publication status, (2) the 3S process(es) addressed and (3) their jurisdiction of publication. Second, we conducted a thematic analysis of the data. Three analytical themes were selected by the CFHI based on their organisational needs and priorities, as follows: (1) 3S definitions, (2) 3S mechanisms and (3) conditions that enable or limit the potential for 3S (Table 1). We should emphasise that, while the definition of ‘mechanisms’ used in this study is supported by Normalisation Process Theory (NPT), NPT was not used as a theoretical lens to extract, analyse and record data specific to the 3S mechanisms. NPT is a sociological approach developed to understand the dynamics of integrating new technologies and innovations, particularly in healthcare contexts; in the present paper, we use NPT to add conceptual traction to our efforts to uncover the mechanisms involved in the 3S of healthcare innovations.

**Table 1:** Description of themes included in the thematic analysis

Theme	Description
Definitions	Statement of the meaning of a word or concept
Mechanisms	Coherence, cognitive participation, collective action, reflexive monitoring through which human agency is expressed [61]
Support conditions	Internal or external factors that enable or limit the potential of an organisational process [62]

Both the descriptive and thematic analyses were performed by a single investigator and were validated through peer-review by stakeholders at CFHI. Following each of three review cycles (submitted December 16th, 2016, February 28th, 2017, and July 19th, 2017), the research team revised and refined the outcomes of the scoping review according to feedback provided by CFHI stakeholders. While it was not among the initial study objectives, recurrent insights emerging from analysis of the data allowed us to inductively identify five key learnings on 3S from which a framework of actionable guidance was developed and submitted to CFHI in the form of a research report (October 12th, 2017). CFHI then created a task force, including the research team and CFHI senior directors, improvement leads and faculty leads, to provide feedback on the framework, which saw multiple iterations before consensus was reached on its final form.

## Results

### Scoping review

**Fig. 1:**
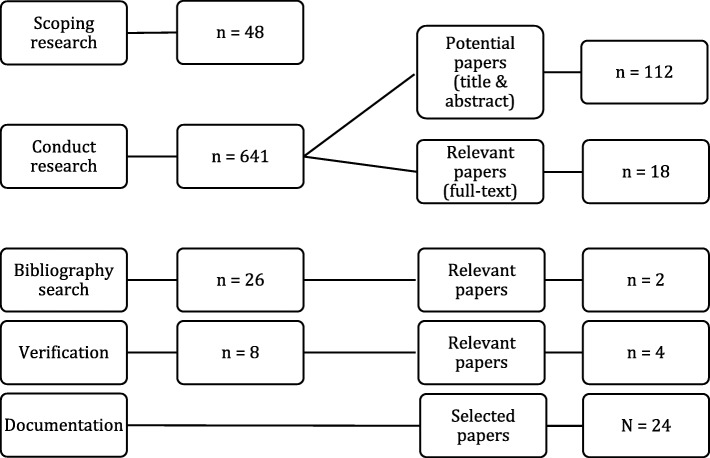
Scoping review search process flow chart

Scoping reviews are useful to answer broad research questions, drawing on a comprehensive literature review to explore the breath of available data produced over a specified time period on a given topic [60]. We performed a scoping review to explore what is known about how to spread, sustain and scale innovations in healthcare. The search and selection process illustrated in Fig. 1 resulted in the inclusion of 24 papers. Of the 24, 15 were peer-reviewed articles and 9 were grey literature publications. The study designs of the peer-reviewed papers included systematic reviews (*n* = 3), case studies (*n* = 3), scoping reviews (*n* = 2), narrative review (*n* = 1), qualitative grounded theory (*n* = 1), longitudinal ethnography (*n* = 1), Delphi technique (*n* = 1) and others (*n* = 3). Most of the scientific and grey literature was informed by sociological, organisational and health sciences disciplines. Overall, the literature mainly focussed on the scale of healthcare innovations (*n* = 7), their sustainability (*n* = 4), spread (*n* = 4), or spread and scale (n = 4), or spread and sustainability (*n* = 4), with only one paper addressing all 3S components. In terms of jurisdiction, most studies were conducted in the United Kingdom (*n* = 10), followed by Australia (*n* = 4), Canada (*n* = 4), the United States (*n* = 3), New Zealand (*n* = 1), the Netherlands (*n* = 1) and Kenya (*n* = 1).

### Descriptive analysis

Descriptive analysis aimed to categorise peer-reviewed articles (*n* = 15) and grey-literature publications (*n* = 9) included in the final selection. Tables 2 and 3 present the data extracted from peer-reviewed articles and grey-literature publications, respectively.

**Table 2:** Key findings from peer-reviewed articles

Authors/title/method	Research question/aim	Main process(es)	Definitions	Mechanisms involved in spread/scale-up/sustainability	Factors that facilitate or impede spread/scale-up/sustainability
Greenhalgh et al., Diffusion of innovations in service organizations: systematic review and recommendations (United Kingdom) [63]	Propose an evidence-based conceptual framework for sustaining innovations	Spread Sustainability	Diffusion: passive spread of innovation Sustainability: making an innovation routine until it reaches obsolescence	Natural, emergent Social Technical Managerial	Innovation system fit Receptive capacity Support and advocacy Dedicated time and resources Opinion leaders and champions Receptive context Interorganisational network, collaboration and boundary spanners
Ferlie et al., The non-spread of innovations: the mediating role of professional (United Kingdom) [64] Two qualitative studies drawing on eight comparative and longitudinal case studies of innovation careers (healthcare and other)	Develops a new theory to explain barriers to spread by asking (1) Are innovation pathways in healthcare linear or messy? (2) Is robust scientific evidence sufficient to lead to successful diffusion? (3) What impact does greater innovation complexity have?	Spread	Spread of healthcare innovations: a slow, complex and contested process, which can be enacted within and across geographical, professional and sectoral boundaries	Crossing boundaries Spreading innovation in multi-professional organisations as a non-linear process	Social and cognitive boundaries between professionals
Slaghuis et al., A framework and a measurement instrument for sustainability of work practices in long-term care (Netherlands) [65]	Develop a theoretical framework and measurement instrument for sustainability	Sustainability	Sustainability: a dynamic process in which actors in a targeted work practice develop and/or adapt organisational routines to a new work method Lacks a theoretical definition and conceptualisation	Routinising Institutionalising	Fit between innovation and work practices, internal structures and dynamics, organisational contexts and institutional orders Dynamism of the process Interorganisational relationships
Norton et al., A stakeholder-driven agenda for advancing thescience and practice of scale-up and spread in health (United States of America) [66] Recommendations from a state-of-the-art conference and follow-up activity to operationalise and prioritise recommendations	Identify gaps and galvanise interest and activity in scale-up and spread of effective health programmes	Spread Scale	Spread and scale: interchangeable terms – deliberate efforts to increase the impact of innovations successfully tested in pilot or experimental projects to benefit more people and to foster policy and programme development on a lasting basis Lack universally accepted definitions	Tracking and sharing information regarding ongoing policy, practice and research in scale-up supported by database and means (e.g. email groups, conference calls, meetings); evidence on when, where and how particular methods are more or less effective, and standardised measures of scale-up and spread	Institutional Review Board regulations of healthcare organisations and systems Funding opportunities and financial incentives to support spread and scale Human resources, capacity and expertise Learning activities that link stakeholders together to share new concepts, critique ongoing scale-up activities Real-time collection of qualitative and quantitative data to guide ongoing adaptations
Lanham et al., How complexity science can inform scale-up and spread in health care: Understanding the role of self-organization in variation across local contexts (Kenya) [24]	Examine the role of self-organisation in the scale-up and spread of effective healthcare practices	Scale-up Spread	Scale-up and spread: efforts (concept, process or practice) to disseminate and implement a successful intervention across systems	Sense-making and interdependencies among stakeholders can facilitate self-organisation processes that increase the probability of spreading effective practices across diverse settings, while acknowledging unpredictability	Understanding of how local context shapes intervention implementation in healthcare contexts Recognition of challenges of behaviour change in healthcare delivery Infrastructure Real-time insights Focus on reproducing interventions with total fidelity, overlooking the unique attributes of local contexts Assumption that innovation is static during the adoption process Connectedness or interrelatedness among project team members
Ploeg et al., Spreading and sustaining best practices for home care of older adults: a grounded theory study (Canada) [67] Development of research-based model	What is the process used to spread best practices related to caring for older adults within home care agencies? What factors influence spread or non-spread?	Spread Scale-up	Spread: process through which new working methods developed in one setting are adopted, perhaps with appropriate modifications, in other organisational contexts Scale-up: no definition mentioned Absence of widely agreed definitions of the terms ‘spread’ and ‘scale-up’	Committing to change Implementing on a small scale, adapting locally, addressing potential barriers Spreading internally to multiple users and sites, then disseminating externally along pathways that are elusive and non-linear with erratic, circular or abrupt processes	Passionate and committed leadership: project leads, champions, managers and steering committees Manager turnover Time and resources Feedback to see benefits
Brewster et al., Integrating new practices: a qualitative study of how hospital innovations become routine (United States of America) [68] Study of hospitals participating in the STAAR initiative in Massachusetts, Michigan (2009–2013)	Examine the process of integrating newly adopted practices into routine hospital operations in order to characterise the mechanisms through which integration occurs	Sustainability	None mentioned	Integrating mechanisms that correspond to different innovation characteristics Innovations that are intrinsically rewarding to the staff, by making their jobs easier or more gratifying, become integrated through shifts in attitudes and norms over time. Innovations with lower innovation-value fit require a different set of integrating mechanisms. Automation of innovation decouples the innovation from staff behaviour when staff did not perceive benefits to themselves Careful monitoring, proactive reminders and problem solving	Visible improvements in outcomes and concrete benefits to staff Passive or active resistance Organisational commitment signalled by senior leaders Continuity of key personnel who can train others and sustain effort while more permanent integrating mechanisms began to work Revised performance standards
Milat et al. (2015) Narrative review of models and success factors for scaling up public health interventions (Australia) Synthesise evidence on scaling up public health interventions into population-wide policy and practice	Define and describe frameworks, processes and methods of scaling up public health initiatives	Scale-up	Scale-up: process by which health interventions shown to be efficacious on a small scale and or under controlled conditions are expanded under real world conditions into broader policy or practice The terms scaling up and scalability have been applied in different ways and contexts with little consistency	Costing and economic modelling of intervention approaches Monitoring implementation of innovation based on data that is linked to decision-making throughout the scaling up process, and a range of implementers and the target community are involved in tailoring the scaled-up approach to the local context	Systematic use of evidence Simplicity of the intervention Ease with which individual intervention components are understood and adopted by key stakeholders and target audiences Infrastructure to support implementation, monitoring and evaluation Political will Clear strategy and strong advocacy Strong leadership and governance Participatory approaches and active engagement of a range of implementers and of the target community
Milat et al. (2014) Increasing the scale and adoption of population health interventions: experiences and perspectives of policy makers, practitioners, and researchers (Australia) Delphi technique	Articulate the processes of how decisions to scale up interventions are made, the role of evidence, and contribution of different professional groups Present perspectives of senior researchers and policy-makers regarding concepts of ‘scaling Generate an agreed definition of ‘scalability’ Identify intervention and research design factors perceived to increase the potential for interventions to be ‘scaled up’	Scale-up	Scalability: the ability of a system, network or process, to handle growing amounts of work in a graceful manner Scale has not been adequately defined in the health promotion literature	Focussing on individual scalability considerations will vary according to intervention attributes, context and the stage of an intervention’s strategic development	Effectiveness, reach and adoption Workforce, technical and organisational resources required Cost considerations Intervention delivery Contextual factors Appropriate evaluation approaches
Milat et al., A guide to scaling up population health interventions (Australia) [69] Systematic review and Delphi technique to offer a four-step guide to scale up an innovation	Develop a guide on how to scale up health interventions, balancing desirability and feasibility	Scale-up	Scale-up: deliberate efforts to increase the impact of successfully tested health interventions to benefit more people and to foster policy and programme development on a lasting basis	Assessing the suitability of the intervention for scaling up includes effectiveness, potential reach and adoption, alignment with the strategic context, acceptability and feasibility Planning scale-up while outlining a vision and compelling case for action, determines who could be involved and what their role will be, considers options for evaluation and monitoring, estimates resources required, secures resources, and builds a foundation of legitimacy	Effect size of the intervention given that effects are likely to be smaller as they are scaled up Local context and the organisational, financial and human resources Formative evaluation to test appropriateness Acceptability of the scaled-up intervention with the target audience and other stakeholders Resources for specific data collection efforts: evaluation and monitoring efforts to show effectiveness over time, rates of reach and adoption, acceptability, compatibility with existing interventions and costs Validity of performance measures and understanding of the limitations of using performance data to inform decision-making Effort to strengthen organisations Coordinated action and governance
Gupta et al., Promoting development and uptake of health innovations: The Nose to Tail Tool [version 1; referees: 3 approved, 1 approved with reservations] (Canada) [70] Scoping review on development of the Nose to Tail Tool	Identify articles that described the scale-up process conceptually or that described an instance in which a healthcare innovation was scaled up Help stakeholders identify the stage of maturity of their innovation, consider each major stakeholder group and contextual barriers	Scale-up	Scale-up: the expansion and extension of delivery or access to an innovation for all end users in a jurisdiction that will benefit from it	Scale-up requires two steps – first spreading to similar settings (expansion) followed by spreading to different settings (extension) Commonly described stages of scaling innovation: identify the problem; develop the innovation; design, conduct, evaluate the pilot test; implementation planning, implementation and evaluation; test for extensibility; scale-up evaluation and monitoring; institutionalisation	Stage of maturity of the innovation and nature of the innovation (discrete, multicomponent or paradigmatic) Clear view of resources required Clear view of the importance of politics and policy Simultaneous attention to vertical or horizontal spread of innovations Opportunity to redesign the innovation at an early stage or cease work on the project before too much has been invested Testing for extensibility Understanding of the interests of key stakeholders, including innovators, end users and decision-makers The social, physical, regulatory, political and economic environment
Greenhalgh et al., Beyond adoption: a new framework for theorizing and evaluating non-adoption, abandonment, and challenges to the scale-up, spread, and sustainability of health and care technologies (United Kingdom) [13] Longitudinal ethnography and action research across more than 20 organisations	Produce an evidence-based, theory-informed, accessible and usable framework Enable those seeking to design, develop, implement, scale up, spread and sustain technology-supported health or social care programmes to identify and address key challenges in different domains and the interactions between them Inform design of new technology, identify technology that has limited chance of scale-up, plan and roll out a technology plan, and learn from programme failures	Scale-up Spread Sustainability	Scale-up: business as usual locally Spread: transferable to new settings Sustainability: maintained long term through adaptation to context over time	Acting collectively and reflexive monitoring help fill crucial gap between the nuanced, flexible and often unpredictable nature of human activity and what it is possible to deliver technically	Complexity of the innovation, clear view of its value proposition and dependability Complexity of the organisation(s) and the wider (institutional and societal) context: degree of readiness, absorptive capacity Technology fit with existing organisational routines Ability to adapt and evolve over time: interaction and mutual adaptation over time between technology, patient, staff and team, with opportunities for sense making of the innovation Complexity in external issues (financial, governance, regulatory, legal, policy), especially reimbursement Devolved organisational structure (with each department or unit able to make semiautonomous decisions) Organisational slack (spare resources available for new projects) Strong leadership, good managerial relations, a risk-taking climate (staff are rewarded rather than punished for trying things out) Resistance or rejection by intended users Ability to shift to new ways of working, or support the extensive work needed to implement and sustain the change
Lennox et al., What makes a sustainability tool valuable, practical and useful in real world healthcare practice? A mixed methods study on the development of the Long Term Success Tool in Northwest London (United Kingdom) [71] A scoping review, group discussion, stakeholder event, interviews and small pilot project	How do sustainability factors identified in the literature resonate with the experience of those in improvement projects in healthcare? Design and test the usability of the tool with healthcare improvement teams	Sustainability	Sustainability: a dynamic process where staff and others involved have the capacity and capability to monitor and modify activities and interventions in relation to the health benefits they wish to achieve and in response to threats and opportunities that emerge over time Several definitions of sustainability in the literature and little consensus on what constitutes ‘achieving sustainability’	Identifying risks to sustainability can create an environment for team members to receive ongoing feedback, highlight specific actions to be taken and comment on ways to influence sustainability over time Acknowledging that sustainability is a process and not an end point, and does not include a specific time frame	Understanding of the relationship between achieving initial ‘successful’ implementation and achieving long-term sustainability Tool design and content Construct design: adequate coverage of items and clear definitions Practical usefulness in real-world healthcare settings Commitment to and support for the improvement Leadership Team functioning Resources, involvement, skills and capabilities Monitoring for feedback and learning: evidence of benefits Process adaptability and robustness Alignment with organisational culture and priorities Alignment with external political and financial environment
Charif et al., Effective strategies for scaling up evidence-based practices in primary care: a systematic review (Canada) [9]	Identify effective strategies for scaling up evidence-based practices in primary care	Scale-up	Scale up: a systematic approach often used in the context of rolling out a successful local programme to regional, national or international levels The term ‘spread’ is commonly used interchangeably with ‘scale up’ Spread: organic process of the diffusion of a local improvement within a health system There is a lack of consensus within the field regarding terminology	Reporting of both a denominator (number of targeted units) and a numerator (number of units covered by the evidence-based practice), in combination with impact measurements Involving strategies related to human resources (policy-makers/managers, providers, external medical consultants and community healthcare workers), infrastructure (new buildings, linkages between different clinical sites), policy/regulation and financing (paying bonuses to healthcare workers), and patient involvement	Human resources Lack of theories, frameworks or strategies to support implementation
Shaw et al., Studying scale-up and spread as social practice: theoretical introduction and empirical case study (United Kingdom) [12]	At an empirical level, what explains the difficulties with spread and scale-up for a particular technology? At a more theoretical level, what kind of insights can a social practice approach provide that will inform the study of spread and scale-up for technological innovations in health and care more generally?	Scale up Spread	Scale up: increase local usage Spread: extend usage to new localities and settings	Balancing the needs of context-sensitivity with the realities of producing technologies that have potential for mass application Coordinating and stabilising shared practices and routines; adoption of a new technology requires changes in the practices adopted by both professional and lay caregivers, and in particular embedding health and care technologies within sociotechnical networks and through situated knowledge, personal habits and collaborative routines. A technology that ‘works’ for one individual in a particular set of circumstances is unlikely to work in the same way for another in a different set of circumstances	Creativity and compassion to generate individual solutions Human relationships and situated knowledge Deep understanding of the complex and situated nature of technology use Clash between the innovation and the actual social practices of real actors

**Table 3:** Key findings from grey literature publications

Authors/title/method	Research question/aim	Main process(es)	Definitions	Mechanisms involved in spread/scale-up/sustainability	Factors that facilitate or impede spread/scale-up/sustainability
Massoud et al., Framework for spread: From local improvements to system-wide change (United States of America) [72]	Provide a snapshot of the Institute for Healthcare Improvement’s latest thinking and work on spread	Spread	None mentioned	Preparing for spread involves acknowledgement by leadership that the improvement project is a key strategic initiative of the organisation, and designation of both executive sponsorship and day-to-day leadership. The existence of successful sites that are the source of the specific ideas to be spread, as well as evidence that the ideas result in the desired outcomes are important Establishing an aim for spread involves identifying the target population, specific goals and improvements, and a time frame for the effort Developing, executing and refining a spread plan includes communication methods and channels to reach and engage the target population, a measurement system to assess progress in meeting the spread aims, and anticipation of actions needed to embed the changes into the organisation’s operational systems	Characteristics of the innovation Willingness or ability of those making the adoption to try the new ideas Characteristics of the culture and infrastructure of the organisation to support change
Clinical Excellence Commission (2008) Enhancing project spread and sustainability: a companion to the ‘easy guide to clinical practice improvement’ (Australia) [73] The Spread and Sustainability Wheel	Provide helpful tips and practical advice to clinicians and health managers on how to improve and asses the spread and sustainability of clinical practice improvement projects	Spread Sustainability	Spread and sustainability: ensure that recognised improvements are maintained beyond the life of the project, and are extended to other areas of healthcare that would also benefit	None mentioned	Nature of initiative Ownership of initiative: leadership and support at senior level Readiness for improvement Effective relationships Integration into practice Evidence of improvements Local context Staff engagement Incentives Processes of implementation Dedicated resources People with influence
Lomas, Formalised informality: an action plan to spread proven health innovations (New Zealand) [74] Summary of the Action Plan to Improve Innovation Spread	Identify gaps and highlight the actions and actors needed to address these gaps for improved spread of innovation in New Zealand’s health sector	Spread	None mentioned	Coordinating, supporting and integrating the three phases of the innovation chain: production/evaluation, dissemination and adoption Interacting interorganisationally is more effective to spread innovations than focussing on structures	Dedicated resources for innovation exploration and development Focused and coordinated evaluation capacity to identify which innovations are worthwhile Commitment from senior leadership Alignment with policy and political priorities Attention to potential adopters’ needs and their balance of costs and benefits Training programmes on innovation-driven change management for managers and clinicians Time set aside specifically for reflection and experimentation by the workforce Slack resources for new projects Relational capital, networks and face-to-face exchanges between stakeholders: Investment in social interaction, not just structures and technology Historical, cultural and economic (dis)incentives for interorganisational collaboration Porous boundaries between the ideas and action communities Boundary-crossing intra- and interorganisational interaction, reflective time: Incentives and networks for ongoing interaction between innovators, evaluators and implementers Targeted persuasive communication, tailored to different audiences Differentiated and decentralised decision-making Specialised focus of professional knowledge in a teamwork environment Because innovations are characterised by novelty and problem orientation, a barrier to spread is their challenge to the status quo
Health Quality Ontario, Spread Primer (Canada) [7]	Spread in the quality improvement framework	Spread	Spread: the active dissemination of best practices and knowledge about interventions, and the implementation of interventions in every applicable care setting Improvement knowledge generated anywhere in the system becomes common knowledge across the system, leading to improvement action	Developing strategies for spreading improvements from the beginning of the improvement project and start small Sharing accountability for spread and empowering others to lead spread builds commitment to common goals as well as the infrastructure to sustain change Ensuring that improvements and the renewed energy and satisfaction that innovations generate reach all parts of the organisation Using a variety of approaches makes it easy for staff to be receptive and adopt change	Nature of the change induced by the innovation Organisational readiness for change Awareness of change concepts and ideas Applicability of potential changes to new environments Belief that change ideas will result in improvement Taking action to adopt the change Sense of urgency and understanding of unmet needs Team collaboration in designing spread plan Regular review of data on defects and performance
Quality Improvement Hub, The spread and sustainability of quality improvement in healthcare (United Kingdom) [75] Literature review	Increase understanding of the 10 key factors underpinning successful spread and sustainability of quality improvement in NHS Scotland	Spread Sustainability	Spread: when best practice is disseminated consistently and reliably across a whole system and involves the implementation of proven interventions in each applicable care setting Sustainability: when new ways of working and improved outcomes become the norm	Disseminating why the change is needed Ensuring that those involved have a desire to support and participate in the change as well as knowledge of how to bring about the change Implementing new skills and behaviours and redesigning processes to sustain the change	Clarity of benefit Real time data to drive improvement Human factors: understanding of why common errors are happening and then redesigning, with steps to prevent the errors Culture: understand the role of culture on behaviours and ability to deliver improvements Change management: support for people to understand the problem a change is attempting to fix and involve them in designing and testing the solutions Leadership combining technical quality improvement skills with effective interpersonal and relational skills Accessibility, use and sharing of knowledge and resources Engagement of everyone with a vested interest, across all levels and roles, in the improvement team Evaluation to understand how activities, outputs and outcomes link and ensure learning and feedback loops are in place Empowerment of staff, patients and carers
Healthcare Improvement Scotland, Guide on spread and sustainability (United Kingdom) [76] Literature review	Summarise existing resources and key pieces of research around spread and sustainability Propose spread and sustainability framework	Spread Sustainability	Spread: the process of communicating new ideas or innovations outside the original system Sustainability: when new ways of working and improved outcomes become the norm	Increasing awareness of the need for greater attention and activity in scale-up, including research, practice and policy activity Expand capacity for scale-up policy, practice and research Facilitating information exchange, collaboration and use of existing knowledge Developing and applying new approaches for evaluation	Attributes of innovation Attributes of adopters Internal and external contextual factors System readiness Evaluation, adaptation, embeddedness and institutionalisation of innovation
What Works Scotland, Evidence review: scaling-up innovations (United Kingdom) [77]	How can small scale innovation be effectively scaled up to create large scale transformational change? Provide actionable messages on how to scale-up healthcare innovations	Scale-up	Scale-up: Delivering or enacting an innovation in a way that increases the number of people benefiting from it while ensuring the original design and measures are maintained	There is no agreement on which approaches to use or on what constitutes success of scaling-up healthcare innovations Considering both ‘hard’ components like metrics, and ‘soft’ components like sociocultural factors when thinking about scalability Scaling is emotionally, mentally and physically demanding Influencing and advocating for innovation enable buy-in to the innovation and scaling process, as opposed to position and authority Collaborating and networking play pivotal roles in spreading innovations by increasing buy-in from stakeholders and increasing the sharing of resources, knowledge and experience Planning for spread while considering that the non-linear nature of spread means that not all dynamics and consequences of an innovation can be planned for in advance Implementing an innovation should use sufficient flexibility while retaining fidelity to the core components Having multiple and creative ways to assess and evaluate the adoption and implementation of an innovation helps to embed it within the larger system Composing teams to scale innovations should be considered carefully to meet needs and team composition should be reviewed regularly to ensure required skills and competencies	Adequate time and planning Adaptation of strategy to the complexity of the innovation Agreement between stakeholders regarding the intentions and goals of the scale-up process Infrastructure and administrative and technical support Distributed leadership across levels and partners: cross-scale interplay and sharing of power through combining top-down and bottom-up approaches Size and complexity of the innovation and scaling goals Collaboration and networking The innovation narrative Encouragement for change Facility of information exchange, collaboration and use of existing knowledge
NHS Institute for Innovation and Improvement, Sustainability model and guide (United Kingdom) [78] Action research	The NHS Sustainability Model and Guide were developed for use by individuals and teams involved in local improvement initiatives	Sustainability	Sustainability: when new ways of working and improved outcomes become the norm	Using the NHS Sustainability Model and Guide (scoring sheets) to support and monitor sustainability of healthcare innovations	Innovation fit with goals and structure Progress monitoring Adaptability Credibility of evidence Benefits beyond helping patients Staff training, involvement and attitudes Leadership: senior and clinical Organisational infrastructure
Gabriel, Making it big: strategies for scaling social innovations, Nesta (United Kingdom) [79] Stages in developing a scaling strategy	How can social innovators spread their innovations? Help social innovators think through their scaling strategies, reflect on the benefits and challenges of different options, and show how others have tackled these issues	Scale-up	Scale-up: increasing the number of people who benefit from a social innovation	Clarifying social, organisational and personal goals for scaling Establishing what to scale up Choosing a route to scale-up (influence and advise, build a delivery network, form strategic partnerships, grow an organisation to deliver) and gearing up to deliver a scaling strategy	None mentioned

### Thematic analysis

#### Definitions

Our review shows that there are no standardised definitions for the 3S of healthcare innovations. Some authors use the terms spread and sustainability, or spread and scale-up, interchangeably [24, 78]. The 3S can be characterised as social, dynamic, non-linear and unpredictable processes [9, 12, 24, 25, 64], and various sub-concepts associated with 3S add to both the complexity and richness of these processes (Table 4).

**Table 4:** Definitions

Concepts	Definitions	Associated sub-concepts
Spread	The process through which new working methods developed in one setting are adopted, perhaps with appropriate modifications, in other organisational contexts [25, 67]	Dissemination [63] Diffusion [59, 63]
Sustainability	The process through which new working methods, performance enhancements and continuous improvements are maintained for a period appropriate to a given context [25]	Adoption [81] Implementation [82]
Scale	The ambition or process of expanding the coverage of health interventions, but can also refer to increasing the financial, human and capital resources required to expand coverage [83]	Scalability [84] Expandability [70] Fidelity [77] Replication [85, 86]

Spread is commonly defined as both passive and deliberate efforts to communicate and implement an innovation, and usually involves adapting an innovation to a new setting [13, 67, 87]. Although the dualistic nature of ‘passive and deliberate’ efforts can give rise to conceptual tensions, many scholars argue that these opposing characteristics emerge along a continuum from diffusion to dissemination of innovations. Along that continuum, diffusion would be associated with passive efforts, and dissemination would refer to more deliberate actions. While some authors describe spread as iterative, we found no studies that established a sequential relationship or degree of iteration between diffusion, dissemination and adoption through the spread process [9, 12, 13, 15].

Sustainability is commonly defined as what happens when an innovation becomes routinised within an organisation or other setting. Sustainability and implementation are closely related; the primary difference is that implementation is time-limited, while sustainability occurs over an undefined time, allowing actors to continuously learn and reflect on their experimentation [16, 88–90].

Scale-up commonly refers to the process in which the coverage and impact of an innovation are expanded to reach all potential beneficiaries. In that sense, what would most significantly distinguish spread from scale is not the processes involved, but the goal. As mentioned earlier, spread aims to communicate and implement an innovation, and usually involves adapting an innovation to a new setting, while scale focuses more on expanding the range of people who would benefit from a given innovation. It mostly consists of broadening innovations from local settings to wider jurisdictional or policy contexts. The concept of scalability [84], expandability [70], fidelity [77] and replicability [85, 86] are associated with scaling up an innovation.

The common definitions of these terms allude to the importance of balancing preservation of the core elements of an innovation (fidelity) with contextual adjustments (adaptability). Evidence on the scale-up of healthcare innovations and large-scale transformation also emphasises the need to balance ‘hard’ assets (e.g. performance metrics) and ‘soft’ assets (e.g. history, relational background, existing partnerships within a given organisational setting) [9, 24, 66, 77]. The successful scaling of healthcare innovations seems to require a balanced and comprehensive set of resources, including financial, technical, relational and political assets. Building on a comprehensive set of capacities may lead to a more successful and sustainable scaling process.

What remains less clear in the definition of 3S is the role of policy environments and governance capacities in shaping the innovation journey within and across healthcare systems. While several frameworks acknowledge the importance of policy, political context and organisational structure to the progress of innovation in healthcare settings, little is known about the relation between governance capacities, which involve the capacity to implement and monitor policies, and the success of the 3S. Although they are generally described as processes on a continuum with well-delineated phases, the 3S may refer to innovation journeys that reflect the uncertain and contextualised nature of innovations, as well as the iterative and overlapping nature of the 3S.

#### Mechanisms

**Fig. 2:**
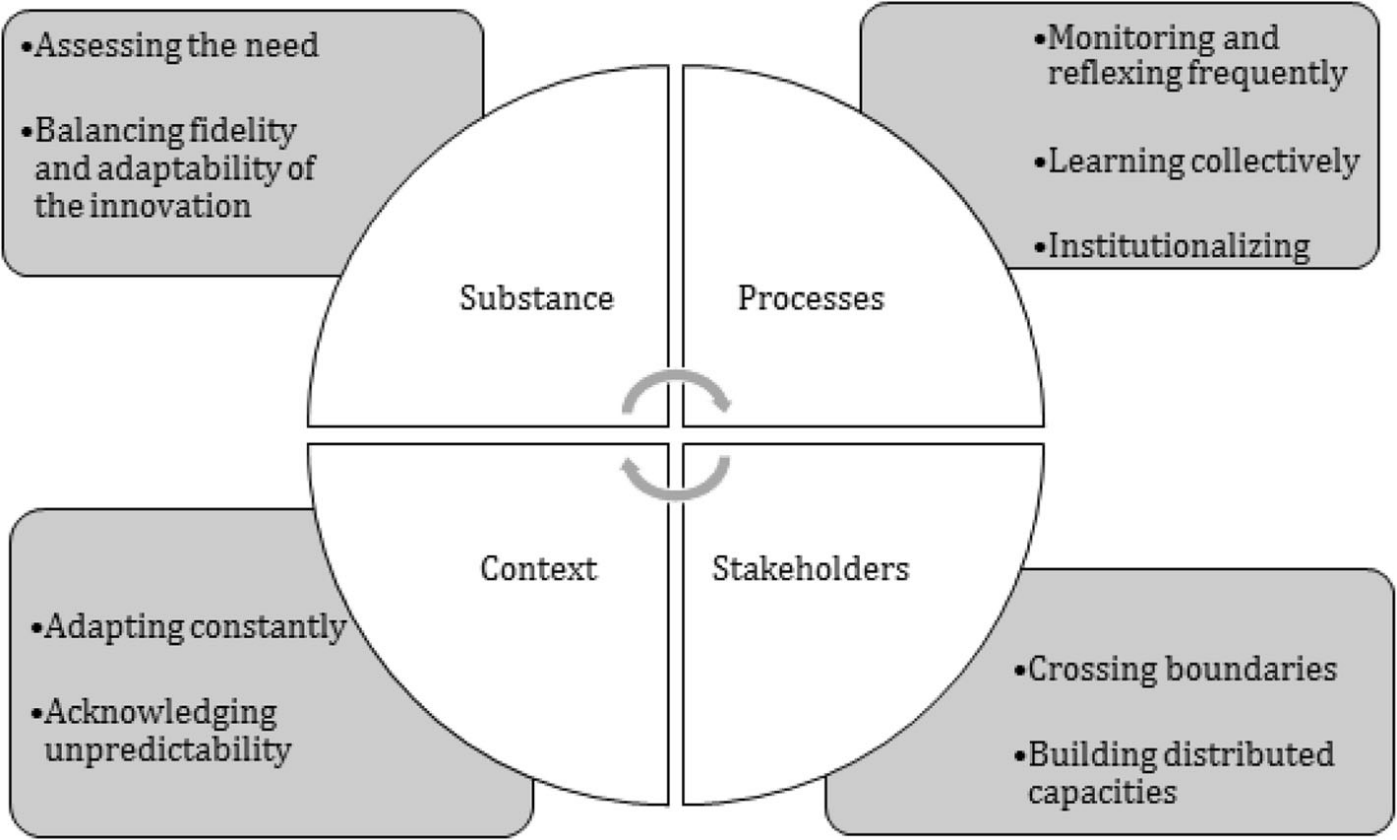
Mechanisms involved in the 3S of healthcare innovations

There are no standardised mechanisms to support the 3S of innovation [66, 91], though many healthcare institutions and agencies have attempted to develop plausible insights into how they might be supported [7, 73–75, 77–79, 92–94]. While the grey literature provides various frameworks and tools, the scientific literature suggests that there is no ‘one size fits all’ approach [1, 13, 25, 87]. Rather, the 3S processes overlap in their operational application, and the mechanisms behind 3S are often described as cutting across these three processes. Based on findings from our scoping review, we argue that 3S mechanisms be categorised along four aspects of the innovation journey, namely substance (innovation), processes, stakeholders and context (Fig. 2).

##### Substance

As argued by Rogers [57, 58, 59], characteristics of the substance of an innovation influence 3S. While the substance of an innovation is variable, the innovation results from successful exploitation of people’s ideas and capacities [91]. Given the diversity of actors, ideas and capacities in healthcare systems and organisations, the source of innovation is dynamic [95]. While healthcare has what Berwick calls a ‘pro-innovation bias’ [96, 97], healthcare innovations are not always appropriate, valuable or feasible. Therefore, actors must engage in a serious assessment of the relative advantage of the innovation not only by patients, but also by providers, managers, policy-makers and sometimes third parties. If the innovation is viewed favourably, the next challenge for its 3S is balancing fidelity and adaptability [25, 98]. This paradox arises from a need for continuous contextual adaptation, without crossing the line beyond which the innovation becomes ‘too different’ to deliver the expected improvement [71, 99, 100]. The literature suggests paying attention to the substance of the innovation, while monitoring outcomes to be sure that 3S generates continuous improvement towards the initial objective [88].

##### Processes

Processes show up in the dynamics underpinning a phenomenon such as the 3S [101–103]. The literature identifies some specific processes associated with spread and sustainability (e.g. diffusing, disseminating, adapting, adopting, implementing), but these are less clear for scale-up [66, 89, 104]. There is a need to identify and understand the cumulative effect of processes associated with sustainability and spread that can support the systemic uptake (scale-up) of innovation.

If we take a broader view of the processes involved in the 3S of healthcare innovations, there is consensus on the fundamental role of frequent monitoring and feedback. These mechanisms seem crucial for maintaining favourable stakeholder perception of the value and feasibility of the innovation over time. Less well-studied is the optimal balance between soft and hard metrics [77]. Use of quantitative data seems to support sustainability [73, 78, 100]. Use of monitoring and feedback for frequent reflection on the outcomes of innovation triggers a collective form of learning, which is associated with better chances of success in 3S [105]. Through collective learning, new collective cognitive products may lead to behavioural changes that foster the institutionalisation of new values, beliefs, norms and organisational practices around the innovation [65, 105]. This is particularly relevant for sustainability, as the innovation becomes an intrinsic part of the organisation or system’s attitudes, norms, beliefs and behaviours.

##### Stakeholders

The complexity of healthcare systems and stakeholders is both a barrier and facilitator to 3S. However, a paradox often appears, where the need to recognise and rely on distributed leadership to support the innovation journey arises in a context of interprofessional and interorganisational boundaries [64, 95, 106]. Consider the strong influence of the distribution of powers between the policy and delivery sides of healthcare systems, seen most obviously in structural hierarchies and accountability relationships [31, 107]. While this reality can sometimes limit the potential to 3S innovations, it can also strengthen 3S when stakeholders cross clinical, organisational, policy and jurisdictional boundaries to create distributed forms of agency [12, 74, 94]. Crossing boundaries increases the scope of capacity-building needed to support and operationalise 3S, fostering continuous improvement in healthcare within and across jurisdictions [108].

##### Context

According to renowned healthcare improvement expert Berwick, “*Researchers who wish to understand how improvement works, and why and when it fails, will never succeed if they regard context as experimental noise and the control of context as a useful design principle*” [96, 97]. In line with Roger’s theoretical take (DIM) on the social nature of diffusing innovations, as well as Shaw et al.’s idea of looking at the 3S of innovation as social practices, Berwick highlights the need to recognise context as an active social ingredient in 3S [109]. The evolution of context itself may bring alignment between adaptation of the innovation and organisational needs and capacities. Though demanding, stakeholders must acknowledge and capitalise on the unpredictability of context, and its influence on the 3S journey [1, 24, 25], to assure that the innovation remains seen as credible, valuable and feasible. Indeed, the success of 3S is dependant on an understanding of context, whether at the individual level, or as manifest in structural elements such as governance, resources, incentives, and accountability or regulations.

#### Enablers and barriers

There is no consensus on the ‘right’ combination of enabling conditions for the 3S of healthcare innovations [75], and little evidence on when, during the 3S journey, they should be mobilised. However, seven enabling factors emerged from our analysis as the most frequently identified and influential (Table 5). Of these, the two most important for potential innovation adopters within healthcare organisations or at the system level are the perceived value and feasibility of the innovation [9, 80, 98, 110, 111]. Indeed, perceptions are embedded in a complex web of other conditions, including the substance of the innovation, leadership, accountability, context, timing, management support and governance. However, a healthcare innovation appears more likely to spread, sustain and scale successfully if stakeholders shift their focus to recognise in these conditions the potential for new collaborations, the development of new capacities, and the empowerment of patients, citizens and providers. New possibilities can emerge from collaborations within and across jurisdictions, a reciprocal mix of top-down, bottom-up and unconventional leadership, and protected time and space for learning, adapting and building innovation capacity [12, 13, 15, 24, 25, 64–67, 69, 70]. We note a gap in evidence on the role of patients, families, citizens, third parties (e.g. research networks) and policy as enabling conditions to 3S.

**Table 5:** Support conditions of the 3S

Support conditions	Enabling	Limiting
Substance (innovation)	Adaptable	Static
Leadership	Distributed	Hierarchical
Accountability	Reciprocal	Unilateral
Context	Absorptive	Tense
Timing and pace of change	Iterative	Linear
Management support	Empowering	Symbolic
Governance	Decentralised	Centralised

## Discussion

**Fig. 3:**
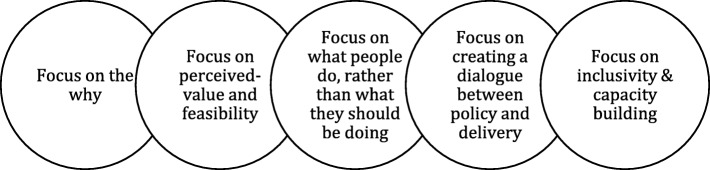
Framework of actionable guidance for 3S across five key focus areas

In this paper, we review scientific and grey literature evidence on the 3S of healthcare innovations to better understand how they work as well as the mechanisms and conditions that either facilitate or hinder 3S. Health systems, supported by various agencies, are paying increasing attention to the problem of the 3S of innovations [13, 18, 81, 84]. While they are not always well supported by evidence or applied appropriately, processes of 3S are powerful engines to propagate these types of innovation. Health systems demonstrate much less capacity to support innovations in models of care or strategies to achieve large-scale improvements. We will look, in this section, at the policy and practical implications derived from analysis of the grey and scientific literature on how to spread, sustain and scale healthcare innovations from local settings to large-scale systems, focusing (1) on the why, (2) on perceived-value and feasibility, (3) on what people do, rather than what they should be doing, (4) on creating a dialogue between policy and delivery, and (5) on inclusivity and capacity-building. We embed these practical implications within a framework of actionable guidance for 3S across five key focus areas (Fig. 3). This framework aims to encourage health system actors to focus on five main components of innovation journeys through the 3S. Our review of the literature finds that values, feasibility, capacity, inclusivity and learning are significant elements in the process of innovation in healthcare organisations. Our framework suggests that there is a complementary relationship between these elements. An integrated perspective that pays attention to each of these components would allow the emergence and identification of significant sources of change across innovation journeys in 3S, from delivery right through to policy. Our findings in this scoping review do not enable us to determine whether different degrees of attention are needed in processes of spread, sustainability and scale. However, given the dynamic, non-linear and sometimes overlapping journeys of the 3S of innovation that can simultaneously cohabitate, we argue that it might be better to support an integrated focus on key elements that intersect and enrich all these processes, rather than invest efforts in trying to dissect their individual paths.

### Focus on the why

An innovation is not an invention, and what is new to some organisations or practitioners may already be very familiar to others. An innovation will have different meanings for different people, which is something that should be valued. Meanings and values that emerge through 3S may challenge usual practices or reveal that an innovation is ill-suited to a given context and consequently result in its rejection. However, the evidence suggests that, if a sufficient number of individuals or organisations have adopted an innovation, it may successfully spread across a system [57]. Given the complexity, dynamism and plurality of healthcare institutions, it appears utopian to expect that the meaning of an innovation remains static over time [112]. Rather than try to propagate a standardised vision of an innovation within a given organisational setting or system, energies should focus on ensuring that everyone involved in or affected by the 3S process can answer why they commit to the innovation; answers will not be the same for everyone [75]. Lags in momentum and interruptions are to be expected along the 3S journey, but it is crucial that stakeholders consider that the innovation adds value to their work and to the quality of care and services they provide to patients [25]. As found by the NHS Scotland Quality Improvement Hub, “*focussing on the why*” ([94], p. 4) involves efforts such as sharing evidence on the relative advantage of the innovation, highlighting promising experiences from other jurisdictions, and monitoring and measuring performance to see improvement.

### Focus on perceived value and feasibility

Innovation is always, to some degree, disruptive [113]. Innovation demands changes in the usual ways of doing things in an organisation or system [114, 115]. We call the efforts to spread, sustain and scale-up innovations ‘innovation work’ to reflect the emotional and behavioural adjustments potential users must make to put an innovation into practice. Further, adjustments reach beyond the level of individual adopters. The implementation of a new model of care requires changes in the roles of professional groups, in the relationships between providers from various sectors, in the financing of care, in regulations and labour contracts, and in the politics that shape care delivery [116]. Any significant innovation is a source of destabilisation and change for practice settings, and requires commitment from influential leaders and the development of policies to promote alignment between attributes of the innovation and existing regulations, thereby mitigating the negative effects of change [34]. Innovation work can be facilitated by support from influential leaders and by policies that promote alignment between the characteristics of the innovation and system functioning and regulations [104, 116]. Given the effort and energy required, the focus of 3S must be on the perceived value and feasibility of innovations for health system actors. Efforts deliberately engaged by organisational actors, especially in disruptive contexts, are significantly motivated by the value they intend to create. The value pursued by health system actors may refer to the ‘quadruple aim’ of improvements in patient experience, population health and the well-being of healthcare teams, along with reductions in cost. However, as discussed earlier in this paper, value can be decontextualised by individuals into what they intrinsically aim to create or maximise for users, families, citizens, colleagues, etc. In highly pluralistic environments such as healthcare organisations, the feasibility of the efforts innovations require appears as a powerful condition to generate and maintain common values among actors. The belief in people that they are equipped and able to contribute to 3S is crucial to maintaining motivation over time [64, 117–120]. Supporting and guiding collective action towards common goals throughout the innovation journeys requires the agility to create complementarities among stakeholders, even as each seeks to bring value to their own work and reinforce each other’s competencies to achieve value.

### Focus on what people do, rather than what they should be doing

Politicians and policy-makers are often impatient to see change and improvement in health systems [104]. They design and adopt policy reforms that often, from the point of view of healthcare providers, involve a wide range of innovations. Providers often must learn to work and collaborate differently to make innovation a reality in their practice setting. They need support to learn new ways of organising work and delivering care. Innovations are not adopted by reorganising people and rules to support, sustain and eventually spread and scale them up. Rather, innovation will become routine practice if providers have time to incorporate new practices into their local context, learning as they do so, and designing an approach that fits well with local needs and capacities [65]. This is one of the more delicate balances to manage in healthcare innovation – the need to leave space for local adaptation and the risk of diluting the strengths of the innovation [1, 121, 122]. It is not realistic to expect managers and policy-makers to support an open agenda for 3S, nor for providers to maintain motivation and commitment without incentives, especially when the innovation’s benefits in improving patients’ health status and care experience is unclear. However, forcing innovation work within a short-term agenda might hinder its potential sustainability [1]. The focus must therefore be on what people do, rather than what they should be doing. One strategy is to adopt management tools that continuously monitor and provide feedback on the ongoing work accomplished by stakeholders, rather than management tools that aim to increase control and coercion over expected work [123]. The more an innovation circulates across a variety of settings and contexts, the more it – and the stakeholders involved – will change [124]. Focussing on what people do, rather than on what they should do, helps to identify the sources of value and issues of feasibility in innovation work. Moreover, this allows us to situate the value and feasibility of innovation in a mechanism to assess and monitor the innovation process, which creates and protects room for adaptation in the innovation, in people and in the system.

### Focus on creating a dialogue between delivery and policy

There is growing recognition of the importance of context in shaping the destiny of innovation. Context is a multi-faceted concept. It can refer to broader policy and political context, and to more micro organisational or clinical contexts. The more diverse the contexts (political, organisational, clinical) an innovation touches, the more it will demand exchanges among a variety of actors [125]. An innovation will navigate these interlinked contexts along its journey from delivery to policy, or from policy to delivery [97]. For example, propagation of a new model of primary care may be influenced by negotiations between medical associations and government. To accommodate the multiplicity of contexts and forms of knowledge in the innovation journey, delivery and policy actors will establish a dialogue to arrive at common views of challenges and opportunities. Facilitating an innovation journey requires more than discussions across groups or organisations. This part of innovation work is essentially relational – the aim is for stakeholders to negotiate a way to move an innovation forward that will take their values and interests into account. Strategies to integrate the values and interests of a wide array of stakeholders may include forums and seminars that enable dialogue and problem solving, as well as informal opportunities for communication and deliberation between actors from all levels, from delivery to policy, who may have different views and interests. Champions of an innovation are often seen as facilitators to bridge the various groups affected by the propagation of an innovation, but let’s think outside the box. Evidence points to benefits from distributed and unconventional (e.g. medical secretaries, support staff, patients and citizens) forms of leadership around the 3S of innovation in healthcare [71]. While there are challenges associated with distributed leadership, such as shared decision-making and governance capacities, the presence of genuine experimenters is crucial to accelerate the impact of the 3S of innovation [106, 126]. Dialogue between delivery and policy bodies during innovation journeys (3S) is a significant condition for increasing value, bringing coherence and creating complementarities among parts of healthcare systems that may challenge the penetration of new ways of thinking and doing.

### Focus on inclusivity and capacity-building

Health systems are driven by the views, values and interests of multiple professional groups and organisations. In such an environment, it is difficult to promote an innovation by decree [127]. The risk of inertia is high and the propagation of innovations that challenge the status quo is slow. Innovations that are minimally or potentially disruptive will be adopted in health systems if they can challenge this inertia. There is a political economy inherent to health systems, and innovations that affect the allocation and circulation of resources or challenge the position of powerful groups will require explicit discussion and strategies to move forward [112]. The focus must therefore be on fostering distributed governance capacities. The involvement of new actors, such as citizens in health policy and patients in the design of care, may provide a strategy for moving forward. However, this may be insufficient on its own – multiple levers for large-scale transformation and improvement are needed. Countervailing powers, such as evidence of the pay-off of innovations, comparison between current practice and the proposed innovation, monitoring and measurement of performance gaps in the system, and dissemination of promising experience in other health systems, may help to challenge the status quo.

#### Strengths and limitations

This study has several strengths and limitations. In terms of strengths, it offers a timely and unique contribution by presenting the state of knowledge, reflected in peer-reviewed and grey literature from various jurisdictions and using a wide range of study designs and methodologies, on how to facilitate the 3S of healthcare innovations. The study used a transparent, rigorous and replicable review process, and was developed collaboratively by researchers and decision-makers (CFHI). It contributes to filling current gaps by providing conceptual and operational guidance to support the spread, sustainability and scale of healthcare innovations within complex policy environments. However, our study presents some limitations. First, the scoping review design did not involve assessing the quality of included papers. Second, given the lack of methodological standards for scoping review designs, some scholars may disagree with our review process, which was supported by Booth’s methodological approach [128]. Lastly, the framework of actionable guidance for 3S across five key focus areas suggested in this paper has not yet undergone empirical validation. Future research should explore and validate the empirical application of the framework to better understand how to facilitate the 3S of healthcare innovations.

## Conclusion

Our review makes it clear that innovation is not a discrete event, but truly a journey. It encourages us to think of innovations as unpredictable and contextualised, which may therefore give rise to multiple journeys that interact and overlap over the course of the 3S. We have summarised five key lessons that can inform the experience of clinicians, managers, policy-makers, patients and citizens with innovations in health systems and, more importantly, can support their actions. These five lessons may constitute the ingredients for what we call ‘innovation work’ in health systems. The paper’s main contribution, in looking at existing work of the 3S of healthcare innovations, is a comprehensive view of the definitions, mechanisms and support conditions involved in 3S. Further research could look more closely at the role of regulations and legislation in the governance of spreading, sustaining and scaling-up healthcare innovations. Integrating research knowledge around policy capacities and innovation may be helpful. Moreover, while we recognise that theoretical contributions have been made to the field of innovation research applied to healthcare contexts, we argue that there is a need for greater consensus on the theoretical definition of what the 3S are and how they proceed. The current consensus gap jeopardises the production of generative empirical studies, leaving scholars to study this process with only fragmented theoretical insights. We invite researchers to pay greater attention to unsuccessful experiences with the 3S of healthcare innovations, which could help to elucidate the challenges involved and lessons learned to inform future initiatives. We consider that further empirical research could adopt realistic evaluation designs in order to uncover the generative mechanisms that expose how innovations are understood to work, by whom and in which circumstances through the unpredictable journeys of spreading, sustaining and scaling [129]. Moreover, realist evaluation could provide theoretical contributions by generating middle-range theories around the 3S of healthcare innovations.

## Data Availability

All data generated or analysed during this study are included in this published article.

## Abbreviations

3S: spread, sustainability, scale
CFHI: Canadian Foundation for Healthcare Improvement
DIM: Diffusion of Innovations Model
NHS: National Health Service
NPT: Normalisation Process Theory
